# Is knowledge of HIV status associated with sexual behaviours? A fixed effects analysis of a female sex worker cohort in urban Uganda

**DOI:** 10.1002/jia2.25336

**Published:** 2019-07-09

**Authors:** Katrina F Ortblad, Daniel K Musoke, Thomson Ngabirano, Joshua A Salomon, Jessica E Haberer, Margaret McConnell, Catherine E Oldenburg, Till Bärnighausen

**Affiliations:** ^1^ Department of Global Health University of Washington Seattle WA USA; ^2^ International Research Consortium Kampala Uganda; ^3^ Uganda Health Marketing Group Kampala Uganda; ^4^ Department of Global Health and Population Harvard T.H. Chan School of Public Health Boston MA USA; ^5^ Department of Medicine Stanford University Stanford CA USA; ^6^ Department of General Internal Medicine Massachusetts General Hospital Global Health Boston MA USA; ^7^ Francis I. Proctor Foundation University of California San Francisco San Francisco CA USA; ^8^ Department of Ophthalmology University of California San Francisco San Francisco CA USA; ^9^ Department of Epidemiology & Biostatistics University of California San Francisco CA USA; ^10^ Africa Health Research Institute KwaZulu‐Natal South Africa; ^11^ Heidelberg Institute of Public Health University of Heidelberg Heidelberg Germany

**Keywords:** testing, key and vulnerable populations, sex workers, women, Knowledge of HIV status, Uganda, sexual behaviours, condom use

## Abstract

**Introduction:**

Female sex workers (FSWs) have strong economic incentives for sexual risk‐taking behaviour. We test whether knowledge of HIV status affects such behaviours among FSWs.

**Methods:**

We used longitudinal data from a FSW cohort in urban Uganda, which was formed as part of an HIV self‐testing trial with four months of follow‐up. Participants reported perceived knowledge of HIV status, number of clients per average working night, and consistent condom use with clients at baseline, one month, and four months. We measured the association between knowledge of HIV status and FSWs’ sexual behaviours using linear panel regressions with individual fixed effects, controlling for study round and calendar time.

**Results:**

Most of the 960 participants tested for HIV during the observation period (95%) and experienced a change in knowledge of HIV status (71%). Knowledge of HIV status did not affect participants’ number of clients but did affect their consistent condom use. After controlling for individual fixed effects, study round and calendar month, knowledge of HIV‐negative status was associated with a significant increase in consistent condom use by 9.5 percentage points (95% CI 5.2 to 13.5, *p *<* *0.001), while knowledge of HIV‐positive status was not associated with a significant change in consistent condom use (2.5 percentage points, 95% CI −8.0 to 3.1, *p *=* *0.38).

**Conclusions:**

In urban Uganda, FSWs engaged in safer sex with clients when they perceived that they themselves were not living with HIV. Even in communities with very high HIV prevalence, the majority of the population will test HIV‐negative. Our results thus imply that expansion of HIV testing programmes may serve as a behavioural HIV prevention measure among FSWs.

## 1 Introduction

Knowledge of HIV status is a necessary pre‐condition for biomedical and behavioural HIV interventions that have been proven to reduce the risk of HIV transmission 1, 2, 3, 4, 5, 6, 7, 8, 9, 10, 11, 12. Many governments and international organizations have thus invested heavily in HIV testing 13, 14, including clinic‐based testing 13, home‐based testing 15, 16 and HIV self‐testing 17, 18, 19, so that individuals can initiate treatment as prevention (TasP) 3, 4 or pre‐exposure prophylaxis (PrEP) 7, 8, 20, 21, 22, 23 to prevent HIV transmission or acquisition respectively. The effect of knowledge of HIV status on the sexual behaviours that increase the risk of HIV transmission, however, remains unclear despite numerous studies in diverse populations 24, 25, 26, 27, 28, 29, 30, 31, 32, 33, 34, 35, 36, 37, 38.

Concerns remain that knowledge of HIV status, acquired through HIV testing, may increase sexual behaviours associated with an increased risk of HIV transmission 26, 29, 34. Individuals who test HIV‐negative may believe they are immune from HIV infection because their past HIV risk‐related sexual behaviours have not led to infection 24, 34, while individuals who test HIV‐positive may no longer fear HIV infection 26, 29. Alternatively, acquisition of knowledge of HIV status, related to HIV testing, may decrease sexual risk‐taking behaviours. Individuals who test HIV‐negative may want to protect themselves from HIV acquisition, while individuals who test HIV‐positive may want to prevent transmission to their sexual partners 36, 37.

The majority of previous studies among members of the general population in high HIV prevalence settings found no association between knowledge of HIV‐negative status and individuals’ sexual behaviours 25, 26, 29, 30, 31, 32, 33 and a negative association between knowledge of HIV‐positive status and individuals’ HIV risk‐related sexual behaviours 24, 25, 27, 28, 30, 31, 33. Knowledge of HIV status may have different effects on sexual behaviour among female sex workers (FSWs) in similar settings, because FSWs face strong economic incentives for many sexual partners and the provision of condomless sex 39. Previous studies have found knowledge of HIV‐positive status among FSWs to be associated with increases 35, decreases 36, 37, and no change 38 in HIV risk‐related sexual behaviours.

The existing literature on knowledge of HIV status and sexual behaviour has a number of limitations. To begin with, all previous studies among members of the general population were conducted prior to the emergence of evidence on the efficacy of TasP 3, 4 and PrEP 7, 8. Knowledge of TasP and PrEP may reduce the effect of HIV status knowledge on HIV risk‐related sexual behaviours, because it reduces concerns about HIV transmission and acquisition. Moreover, all previous studies that examined the relationship between HIV status knowledge and sexual behaviour among FSWs used cross‐sectional data and thus do not allow strong causal inferences.

With this study, we aim to substantially strengthen the evidence on the effect of knowledge of HIV status on sexual behaviour among FSWs in the current era. We do so using cohort data, which allows us to use a quasi‐experimental estimation approach: individual fixed effects estimation. The individual fixed effects remove potential bias due to all individual‐level confounding factors – whether observed or unobserved – that are stable over the study period of four months. In addition, we control for study round and calendar time, adding further causal strength by removing bias due to study round‐ and time‐varying confounding factors.

Understanding the effect of knowledge of HIV status on sexual behaviours among FSWs is important for policy and practice. In general, it can support the design of interventions to ensure that HIV testing initiatives are best leveraged for sexual behaviour risk reduction. It can also specifically help ensure that HIV testing interventions targeted at FSWs decrease, rather than increase, the risk of HIV transmission between FSWs and their clients.

## 2 Methods

### 2.1 Participant cohort

The cohort of FSWs we are using for the analyses in this paper was formed as part of a three‐arm cluster‐randomized controlled trial of HIV self‐testing delivery models in urban Uganda, which was carried out from 2016 to 2017 19. The trial is registered in the registry of the US National Library of Medicine, ClinicalTrials.gov (registration number: NCT02846402). The primary pre‐registered outcomes for this trial were any HIV testing at one month and at four months. Participants were thus followed for four months.

Over the duration of the trial, participants had ample opportunity for HIV testing. All participants were referred to free standard of care testing services by a FSW peer educator. Participants in the HIV self‐testing intervention arms received either an HIV self‐test kit or a coupon for an HIV self‐test kit from their peer educator shortly after enrolment and again three months later. We used the OraQuick Rapid HIV‐1/2 Antibody Tests (OraSure Technologies, Bethlehem, PA) in this study, which have 93% sensitivity and 99% specificity compared to standard‐of‐care blood‐based HIV testing 40. All participants were trained by an FSW peer educator on how to use and interpret the HIV self‐test results. Participants who reported testing HIV‐positive (via self‐testing or clinic‐based testing) during the four‐month duration of the study were counselled by research assistants and peer educators on the effectiveness and free availability of HIV treatment, and were immediately referred to the nearest clinic where HIV treatment was available. Detailed methods for this trial have been published elsewhere 19.

Ethical approval for the trial was granted by the institutional review board at the Harvard T.H. Chan School of Public Health and the Mildmay Uganda Research Ethics Committee. We obtained written informed consent from all participants.

### 2.2 Study settings

Kampala, the capital city of Uganda, is a major centre of commerce in East Africa. Many migrants move to Kampala to pursue economic opportunities including sex work. There are approximately 13,000 FSWs in Kampala; one in three is estimated to be living with HIV 41. The Ugandan Ministry of Health (MOH) prioritizes FSWs for the delivery of health inventions through the Most at Risk Populations Initiative (MARPI) 42, a service that is commonly used among members of this population.

### 2.3 Eligibility

Participants were recruited for the trial by FSW peer educators and assessed for eligibility by research assistants 19. Eligible participants were: 18 years of age or older, exchanged sex for money or goods at least once in the past month, and reported never testing for HIV or testing HIV‐negative at their last test (more than three months prior) 19.

### 2.4 Data collection

Participants completed three study rounds of data collection: at baseline, one month, and four months. The timing of these rounds was the same for all participants. All data were collected electronically (CommCare, Dimagi Inc, Cambridge, MA) by research assistants in face‐to‐face interviews at private locations selected by participants (e.g. empty bar, home, guest house).

### 2.5 Sexual behaviour outcomes

We measured two sexual behaviour outcomes: (i) participants’ number of clients on an average working night and (ii) participants’ consistent condom use with clients. At all study rounds participants were asked to report the number of clients they have sex with on an average working night and the number of clients with whom they use a condom. If participants reported using condoms with all their clients, their condom use was categorized as consistent.

### 2.6 Knowledge of HIV status

We categorized participants’ knowledge of HIV status into three states: (i) knowledge of HIV‐negative status, (ii) knowledge of unknown HIV status, and (iii) knowledge of HIV‐positive status. The knowledge we refer to is the perception of HIV status. We chose this measurement for knowledge of HIV status because it is the perception of HIV status that will primarily determine sexual behaviour. For instance, if a FSW is in fact HIV‐negative but believes that she is HIV‐positive, it is the belief in her HIV status that will guide her behavioural choices rather than the biological status. Pathways through which perceived knowledge of HIV status are likely to affect sexual behaviour include emotions and considerations such as “I am HIV‐positive and thus need to protect my HIV‐negative partners” or “I am already HIV‐positive and thus do not need to protect myself.”

In this study, participants’ self‐perceived knowledge of HIV status may not match the results of their most recent HIV test for a number of reasons including, (i) sexual encounters after the most recent HIV test, (ii) mistrust of a new HIV testing technology (i.e. self‐testing), (iii) uncertainty surrounding their own ability to correctly interpret HIV self‐test results, and (iv) outside interventions intended to cure individuals of HIV (e.g. religious interventions, which are common in the region) 43, 44, 45.

To capture participants’ knowledge of HIV status, at each study round we asked participants to estimate the likelihood that they were currently living with HIV using a 10‐rung ladder scale – an approach adapted from a previous study that measured participants’ perceived risk of HIV acquisition 46. Participants’ responses largely lumped around 1, 5 and 10, and thus we categorized participants’ knowledge of HIV status at each study round as HIV‐negative (rungs 1 to 3), unknown (rungs 4 to 7) or HIV‐positive (rungs 8 to 10).

### 2.7 Covariates

We measured sociodemographic characteristics at baseline. At each study round participants reported recent HIV testing (past three months at baseline, past one month at one month, and past one month at four months) and the results of their most recent HIV test. Our electronic data collection platform automatically captured the study round (e.g. baseline, one month, four months) and calendar month of observation. Because of the spacing of the study rounds, there were some calendar months between the second and last study round when no data were collected. At four months, all participants who gave consent completed a blood‐based rapid HIV test.

### 2.8 Statistical analysis

We used individual fixed effects analysis of longitudinal data to estimate the association between participants' knowledge of HIV status and sexual behaviours. Individual fixed effects estimation controls for all observed and unobserved confounders that do not vary over time within an individual 47. Thus, our estimations are based on the within‐individual changes we observed over the study period. Examples of confounding factors that the individual fixed effects control for – because they cannot, or are very unlikely to, vary over the four‐month study period – include genetic make‐up, birth order, place of birth, ethnicity, language, stable sociodemographic characteristics (such as place of residence and educational attainment), fundamental character traits (such as extraversion or conscientiousness), and stable self‐evaluations (such as locus of control and self‐efficacy). Individual‐level fixed effects estimation thus allows for relatively strong causal inference 48.

However, individual fixed effects do not control for confounders that vary over time within an individual. We thus added two time‐varying factors, study round and calendar month, as independent variables. The study round fixed effects control for confounders that may have changed with study round in similar ways across participants. Such confounders may include changes in social desirability bias due to participation in this study and associations between HIV status knowledge and sexual behaviour that arise because participants in the study are encouraged to both test for HIV and to reduce HIV risk‐related sexual behaviour. The calendar month fixed effects then control for time‐varying changes, such as social marketing campaigns, health system reforms or general underlying societal trends, which affect all participants. To maximize the statistical efficiency of our analyses, we selected the category with the greatest number of observations as the reference for each of these variables 49: baseline round of data collection (for round of data collection) and the month of December (for calendar month).

We used linear panel regression for estimation. Because peer educators were involved in the recruitment of participants and trained participants on how to interpret HIV self‐test results, we adjusted our standard errors for clustering at the level of the peer educator.

To understand how the direction of change in HIV status knowledge is associated with changes in participants’ sexual behaviours, we conducted a number of sub‐group analyses. We divided participants into sub‐groups based on their knowledge of HIV status (HIV‐negative, unknown, HIV‐positive) and sexual behaviours (low risk and high risk) at baseline. By dividing participants by their knowledge of HIV status at baseline, we can better understand how specific changes in knowledge of HIV status from prior knowledge states may be differentially associated with changes in sexual behaviours. For example, a change in knowledge of status from HIV‐negative to HIV‐positive may lead to different sexual behaviours than a change in knowledge of HIV status from unknown to HIV‐positive. We then further divided participants by HIV risk‐related sexual behaviours at baseline (specific to the sexual behaviour outcome of interest: < vs. ≥ baseline median number of clients, and consistent vs. inconsistent condom use with clients), because we can only observe changes in sexual behaviours among participants that are able to change their sexual behaviours from baseline. Examples of directional changes in sexual behaviour following a particular directional change in HIV status knowledge include (i) FSWs who learn that they are HIV‐negative decreasing HIV risk‐related sexual behaviours and (ii) FSWs who learn that they are HIV‐positive increasing HIV risk‐related sexual behaviours. For all sub‐group analyses, we used linear panel regressions with individual fixed effects, controlling for study round and calendar month. We adjusted standard errors for clustering at the level of the peer educator.

We used Stata 13.1 (StataCorp, College Station, TX) for all analyses in this study.

## 3 Results

### 3.1 Participants

From October to November 2016, 1587 potential participants were phone‐screened for eligibility, of which 997 were invited for in‐person eligibility assessment, and 960 were enrolled. Some of the most common reasons for study exclusion were reported HIV testing within the past three months (52%, 325/627) and self‐reported HIV‐positive status (43%, 268/627).

The descriptive characteristics and sexual behaviours of the 960 study participants at baseline are shown in Table 1. The median age of participants was 28 years (interquartile range (IQR) 24 to 32). At enrolment, almost all participants (94%) had tested for HIV at least once, but only three participants (0.3%) had tested for HIV in the past three months. Participants reported earning almost three times as much for a sex act without a condom (mean $9.94, standard deviation (SD) $11.46) compared to a sex act with a condom (mean $3.24, SD $3.31). Participants reported a mean of 5.9 on an average working night (SD 3.8) and 60% of participants reported consistent condom use with clients.

**Table 1:** Participant characteristics at baseline

Age (med, IQR)	28 (24 to 32)
Education	
No formal	79/960 (8.2%)
Primary/junior	437/960 (45.5%)
Secondary	423/960 (44.1%)
Vocational	8/960 (0.8%)
Tertiary	13/960 (1.4%)
Monthly income, USD^a^^,^ b	
No income	5/955 (0.5%)
<$30	190/955 (19.9%)
$30‐$60	332/955 (34.8%)
$60‐$125	328/955 (34.4%)
>$125	99/955 (10.4%)
Timing of last HIV test	
0 to 3 months	3/960 (0.3%)
>3 to 6 months	351/960 (36.6%)
>6 to 12 months	280/960 (19.3%)
>12 to 24 months	156/960 (16.3%)
>24 months	114/960 (11.9%)
Never tested	56/960 (5.9%)
Of 10 clients, # think are HIV‐positive (med, IQR)	7 (5 to 9)
Price for vaginal sex, USD^a^ (mean, SD)	
With a condom	$3.24 ($3.31)
Without a condom	$9.94 ($11.46)
Number of clients/average night (mean, SD)	5.9 (3.8)
Consistent condom use with clients^c^	569/957 (59.5%)
Tested for HIV, since the start of the study^d^^,^ e	
1 month	759/925 (82.0%)
4 months	812/861 (94.3%)

Med, median; IQR, interquartile range; SD, standard deviation.

^a^Price categories in US dollars (USD); October 10th, 2016 exchange rate (1 USD = 3363.85 Ugandan Shillings); ^b^variations in denominators attributable to participants choosing not to respond to particular questions; ^c^defined as not using a condom with at least one client on an average working night; ^d^all characteristics and behaviours measured at baseline with the exception of testing for HIV since the start of the study; ^e^loss to follow‐up was 4% (35/960) at one month and 10% (99/960) at four months.

Loss to follow‐up was low in this cohort 19. At one month, 96% (925/960) of enrolled participants were retained in the study and at four months, 90% (861/960) of enrolled participants were retained in the study 19. Almost all participants reported testing for HIV at least once over the study duration (82% of participants at one month and 94% of participants at four months).

### 3.2 Knowledge of HIV status

**Figure 1:**
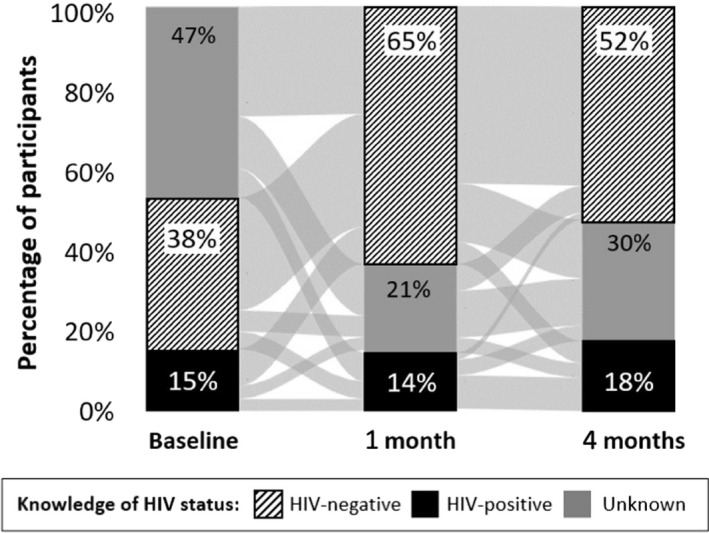
Participants’ knowledge of HIV status at baseline, one month, and four months HIV‐negative status knowledge (black stripes); HIV‐positive status knowledge (black); HIV status knowledge unknown (grey). The light grey lines between the bars show the flows of participants across the different categories of knowledge of HIV status between the three study rounds.

Figure 1 shows participants’ knowledge of HIV status and how it changed over the four‐month duration of the trial. At baseline, nearly half (47%, 444/953) of participants reported knowledge of unknown HIV status; however, by four months, the majority of participants (52%, 445/858) reported knowledge of HIV‐negative status (denominators subject to incomplete reporting). Because our knowledge of HIV status measurement is self‐perceived, changes in knowledge of HIV status may be a result of new information acquired through HIV testing, a new HIV risk encounter following testing, or an outside intervention (e.g. religious encounter) 43, 44, 45. Among participants who participated in all three study rounds, the vast majority (71%, 573/812) changed their knowledge of HIV status over the duration of the trial.

At four months, 92% (340/368) of participants who reported knowledge of HIV‐negative status tested HIV‐negative in a blood‐based rapid test, while 83% (104/126) of participants who reported knowledge of HIV‐positive status tested HIV‐positive in a blood‐based rapid test.

### 3.3 Knowledge of HIV status and sexual behaviours

**Figure 2:**
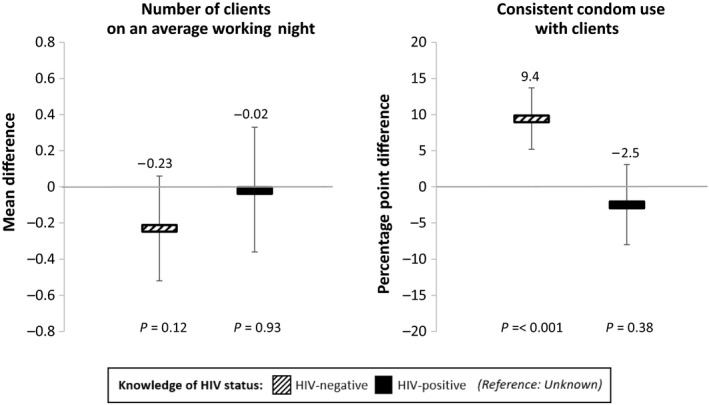
The association between FSWs’ knowledge of HIV status and sexual behaviours with clients The associations between knowledge of HIV status and sexual behaviours (number of clients per average working night and consistent condom use) were measured using linear panel regressions with individual fixed effects, controlling for study round and calendar month. Standard errors are adjusted for clustering at the level of the peer educator. Number of clients per average working night: the bars show the mean differences in the number of clients between (i) those with knowledge of HIV‐negative status and those with knowledge of unknown HIV status (black striped bars) and (ii) those with knowledge of HIV‐positive status and those with knowledge of unknown HIV status (black bars). Consistent condom use: the bars show the average percentage point differences in the probability of consistent condom use between (i) those with knowledge of HIV‐negative status and those with knowledge of unknown HIV status (black striped bars) and (ii) those with knowledge of HIV‐positive status and those with knowledge of unknown HIV status (black bars). The vertical lines indicate the 95% confidence intervals.

Figure 2 shows the association between participants’ knowledge of HIV status and sexual behaviours with clients, when we control for individual fixed effects, study round, and calendar month. In urban Uganda, knowledge of HIV status was not associated with participants’ number of clients per average working night, but it was associated with participants’ condom use with clients. Knowledge of HIV‐negative status was significantly associated with an increase in participants’ consistent condom use with clients. Compared to participants with knowledge of unknown HIV status, consistent condom use was 9.4 percentage points (95% confidence interval (CI) 5.2 to 13.7 to, *p *<* *0.001) higher among participants with knowledge of HIV‐negative status, and 2.5 percentage points (95% CI −8.0 to 3.1, *p *=* *0.38) lower among participants with knowledge of HIV‐positive status. Knowledge of HIV‐positive status, however, was not significantly associated with participants’ condom use with clients.

We conducted four sensitivity analyses to confirm the association between participants’ knowledge of HIV status and sexual behaviours with clients. First, we tested the robustness of our linear regressions with Poisson regressions for number of clients per average working night and logistic regressions for consistent condom use with clients. Second, we tested the influence of adjusting for time‐varying confounders (i.e. round of data collection and calendar month) in our linear regressions by removing these variables from our analyses and running unadjusted models. Third, we tested the robustness of our measure of knowledge of HIV status. Instead of three categories of status knowledge, we used the full 10‐point scale that we used to elicit this information. We used ladder rung 5 as a reference category for these analyses, because it contained the greatest number of observations and thus improved the efficiency of our estimations 49. Fourth, we measured the association between knowledge of HIV status and sexual behaviour among participants who reported any HIV testing over the four‐month duration of the study. We included this analysis to examine whether associations differed systematically among those who were most likely to have objectively improved their knowledge of HIV status with recent HIV testing. For all sensitivity analyses, we used linear panel regressions (with the exception of the first analyses) with individual fixed effects and we adjusted the standard errors for clustering at the level of the peer educator; all but the second set of analyses adjusted for the same time‐varying confounders as in the main analyses (e.g. study round and calendar month).

The findings from our main analyses remained consistent in all four sensitivity analyses (results shown in the Table S1). Knowledge of HIV‐negative status was significantly associated with an increase in participants’ consistent condom use with clients (i) when we used logistic regressions (odds ratio: 4.47, 95% CI 2.27 to 8.78, *p *<* *0.001), (ii) when we used unadjusted linear regressions (percentage point difference: 0.16, 95% CI 0.12 to 0.20, *p *<* *0.001), (iii) when we used a 10‐point HIV knowledge scale (percentage point difference for rung 1 vs. rung 5: 8.2, 95% CI 2.1 to 14.2, *p *=* *0.008; rung 2 vs. rung 5: 13.4, 95% CI 5.9 to 20.8, *p *=* *0.001; rung 3 vs. rung 5: 8.7, 95% CI 3.0 to 14.4, *p *=* *0.003), and (iv) when we limited our sample to participants who reported HIV testing since the start of the study (percentage point difference: 9.3, 95% CI 5.1 to 13.6, *p *<* *0.001). Additionally, knowledge of HIV‐negative status significantly decreased participants’ number of clients on an average working night in the sensitivity analysis with unadjusted linear regressions (ii), but none of the other sensitivity analyses: (i), (iii), (iv). Knowledge of HIV‐positive status was not significantly associated with participants’ condom use or number of clients on an average working night in sensitivity analyses (i) to (iv).

### 3.4 Sexual behaviour changes associated with specific directions of change in knowledge of HIV status

**Figure 3:**
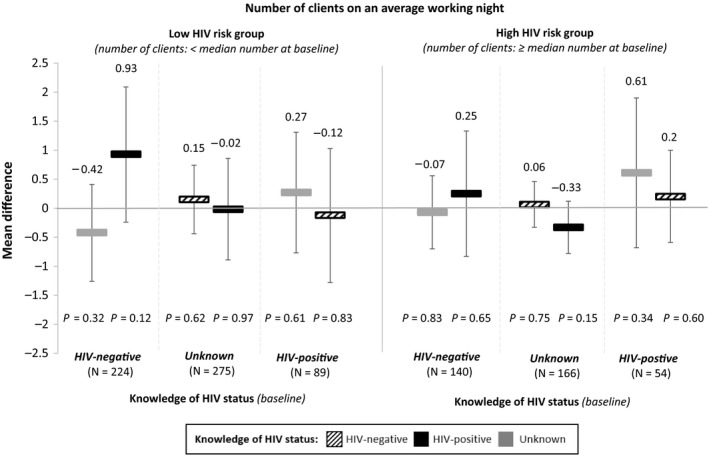
The association between changes in FSWs’ knowledge of HIV status and number of clients from baseline knowledge and sexual behaviours For these sub‐group analyses, participants were sub‐divided by their knowledge of HIV status at baseline and their sexual behaviours at baseline (i.e. low risk vs. high risk). The reference for each sub‐group is participants’ knowledge of HIV status at baseline. The associations between participants’ changing knowledge of HIV and number of clients on an average working night were measured using linear panel regressions with individual fixed effects, controlling for study round (baseline, one month, and four months) and calendar month. Standard errors are adjusted for clustering at the level of the peer educator. The bars show the mean differences in the number of clients for participants whose knowledge of HIV status changed from different states at baseline (listed by sub‐group along the x‐axis) to HIV‐negative (black striped bars), HIV‐positive (black bars), or unknown (grey bars). The vertical lines indicate the 95% confidence intervals.

**Figure 4:**
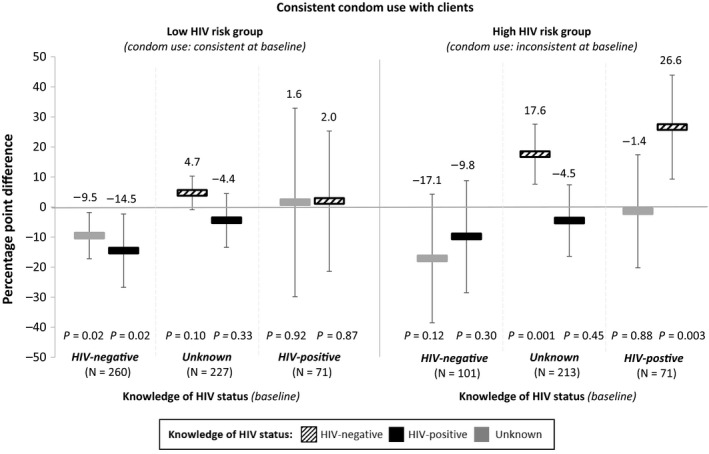
The association between changes in FSWs’ knowledge of HIV status and condom use with clients from baseline knowledge and sexual behaviour For these sub‐group analyses, participants were sub‐divided by their knowledge of HIV status at baseline and their sexual behaviours at baseline (i.e. low risk vs. high risk). The reference for each sub‐group is participants’ knowledge of HIV status at baseline. The associations between participants’ knowledge of HIV status and condom use with clients were measured using linear panel regressions with individual fixed effects, controlling for study round (baseline, one month, and four months) and calendar month. Standard errors are adjusted for clustering at the level of the peer educator. Consistent condom use was defined as not using a condom with at least one client on an average working night. The bars show the average percentage point differences in the probability of consistent condom use for participants whose knowledge of HIV status changed from different states at baseline (listed by sub‐group along the x‐axis) to HIV‐negative (black striped bars), HIV‐positive (black bars), or unknown (grey bars). The vertical lines indicate the 95% confidence intervals.

In sub‐group analyses, we examined how the direction of change in HIV status knowledge is associated with participants’ sexual behaviours. These results, presented in Figures 3 and 4 (the reference for each sub‐group is participants’ knowledge of HIV status at baseline), shed further light on the interpretation of our overall findings. The finding that HIV status knowledge is not associated with the number of clients on an average working night remains robust in all of the sub‐group analyses, which individually measure each of the different directions of change in HIV status knowledge (Figure 3). At the same time, the sub‐group analyses reveal that the finding that knowledge of HIV‐negative status is associated with an increase in consistent condom use is largely driven by one sub‐group: participants in the high HIV risk group (i.e. those who did not consistently use condoms at baseline) with knowledge of either unknown HIV status or HIV‐positive status at baseline (Figure 4).

## 4 Discussion

In urban Uganda, knowledge of HIV status was not associated with FSWs’ number of clients on an average working night, but knowledge of HIV‐negative status was significantly associated with an increase in FSWs’ consistent condom use with clients. This latter finding is different from most of the previous studies on knowledge of HIV status and sexual behaviour, which took place among members of the general population and found that knowledge of HIV‐negative status was not associated with HIV risk‐related sexual behaviours 25, 26, 29, 30, 31, 32, 33. It is particularly encouraging that FSWs in this setting who learn that they are HIV‐negative forgo the opportunity to make almost triple the amount of money for a sex act without a condom compared to a sex act with a condom 50 – presumably to protect themselves from HIV acquisition. Moreover, FSWs in this study did not appear to be making up for this lost income by increasing their number of clients, suggesting that they are prepared to take potentially substantial losses in income for a reduced risk of HIV acquisition.

These results are a powerful indication that expanded and intensified HIV testing services, for instance as part of universal test‐and‐treat policies, can not only serve to identify people for HIV treatment and prevention interventions but can also directly act as a behavioural HIV prevention measure. Another important implication of our findings is that FSWs appear to have a high degree of agency in their sexual relationships, manifesting itself in the power to control condom use with clients 51. This finding is important because it is in contrast to studies carried out two to three decades ago, which reported that FSWs are powerless in their sexual relationships with clients 52 and do not use condoms because they fear violence or abuse 53. Our findings indicating higher degrees of FSW agency could be the result of efforts over the past decades to empower FSWs, which have been supported by the global HIV response 13, 14.

The protective behavioural effect of knowledge of HIV‐negative status in this study is primarily driven by FSWs who did not know their HIV status at baseline. The majority of FSWs who test for HIV will test negative, despite FSWs’ high risk of HIV acquisition 14, 54, 55, 56. While much of the recent emphasis of HIV testing campaigns has been on identifying new individuals living with HIV so they can initiate HIV treatment 57, traditionally HIV testing was very much seen as also serving behaviour change purposes, e.g. as part of voluntary counselling and testing approaches 58, 59. However, for FSWs, evidence on the behavioural effects of HIV testing has hitherto been lacking. Further studies are needed to better understand whether our finding that knowledge of HIV‐negative status on its own decreases sexual risk‐taking in FSW‐client relationships generalizes to FSWs in settings outside of urban Uganda.

In contrast to knowledge of HIV‐negative status, knowledge of HIV‐positive status was associated with a decrease in FSWs’ consistent condom use with clients, but this finding was insignificant in the overall analysis. This finding was significant, however, in the sub‐group analysis of FSWs who consistently used condoms at baseline and whose knowledge of HIV status changed from HIV‐negative at baseline to HIV‐positive. Any decreased condom use among HIV‐positive sex workers with many sexual partners is concerning because this may result in increased HIV transmission. Knowledge of HIV‐positive status may decrease FSWs’ consistent condom use with clients because of their strong economic incentives for unprotected sex. In urban Uganda, FSWs make almost three times as much money for a sex act without a condom than for a sex act with a condom 39. If knowledge of HIV‐positive status eliminates fear of HIV acquisition, FSWs may be more easily persuaded to engage in economically more lucrative unprotected sex with clients – especially if the money is needed to pay for essential goods, such as food or shelter for dependents 60. At the same time, FSWs in Uganda believe that the vast majority of their clients are living with HIV 39 and thus may be unconcerned about infecting their clients. These reasons may explain why the results from our study among FSWs differ from the results of studies in general populations, which have mostly found that knowledge of HIV‐positive status is associated with a reduction in HIV risk‐related sexual behaviours 24, 25, 27, 28, 30, 31, 33, 34.

The potential negative association between knowledge of HIV‐positive status and consistent condom use with clients among some FSWs in Uganda emphasizes the importance of immediate HIV treatment initiation following HIV diagnosis to prevent HIV transmission. To minimize delays in treatment initiation, policy makers could consider home‐ or community‐based delivery of HIV treatment 15, 61 as well as cash transfers for HIV treatment initiation 62, 63. Another solution to preventing the potential negative association between knowledge of HIV‐positive status and FSWs’ HIV risk‐related sexual behaviours may be counselling and education on sexually transmitted infections (STIs). Such education could emphasize the risks associated with condomless, including other STIs and acquiring a strain of HIV that is resistant to antiretroviral drugs. This education could be delivered via FSW peer educators or at the MARPI clinics 42.

Our study has a number of strengths. A first strength is that the study had little loss to follow‐up, limiting the potential for attrition biases. Second, the use of individual fixed effects estimation is a rigorous quasi‐experimental method for determining the effect of knowledge of HIV status on sexual behaviours 48. Since the study period was comparatively short, it is likely that the individual fixed effects in our analyses not only controlled for stable, time‐invariant factors, such as those determined by one's genotype (e.g. height), but also for the wide range of factors that are very unlikely to have changed over four months, such as sociodemographic characteristics.

An important limitation of individual fixed effects estimation, however, is that we cannot rule out that time‐varying confounders that may have biased our results. For instance, it is possible that changes in income that are unrelated to sex work might have affected both HIV testing behaviour (for instance, if financial barriers are important for HIV testing) and consistent condom use with clients (for instance, if higher income reduces the need to sell condomless sex). It is also possible that the different peer educators differentially affected both uptake of HIV testing and social desirability in reporting sexual behaviours, leading to time‐varying confounding of the relationship between knowledge of HIV status and condom use 64. While our results thus substantially bolster the evidence on the relationship between knowledge of HIV status and sexual behaviours, they do not allow causal interpretation of the same strength as the results produced by a randomized controlled trial.

Another limitation of our study is that there may have been measurement error in participants’ reported sexual behaviours. In this study, participants were asked to report the number of sexual clients they have on an average working night. Reporting averages may have been difficult for some participants, especially considering that the majority of participants had either no formal education or only some primary education. Since condom use was measured in relation to number of clients on an average working night, this measurement might reflect more of an attitude towards condom use than a precise estimate. There also may have been some ambiguity about the definition of a sexual client, and depending on the nature of the sex act (which we did not specify in this question) condom use may have been less or more frequent. Despite these limitations, we still saw a significant association between knowledge of HIV‐negative status and participants’ condom use with clients. Future analyses should consider using more precise estimates for number of clients and condom use with clients (e.g. number of clients and condom use within a defined time period).

The participants in our study may also have been more likely to change their sexual behaviours over time compared to other FSWs because of their participation in study‐related activities 65, including four peer educator visits. This may have exaggerated the positive effect of knowledge of HIV‐negative status on FSWs’ consistent condom use with clients, because the peers may have encouraged participants to use condoms to maintain their HIV‐negative status and, additionally, peers distributed condoms to participants at each visit. We also only assessed the relationship between knowledge of HIV status and FSWs’ sexual behaviours with clients. Knowledge of HIV status may have different effects on FSWs’ sex behaviours with non‐commercial partners, because FSWs may care more about the health of romantic partners and may hold different beliefs about a non‐commercial partners’ HIV status.

Participants in our study were followed‐up for a duration of four months. This duration of follow‐up is adequate to determine the short‐term effects of knowledge of HIV status on sexual behaviours. The FSWs in our sample – unlike people in the general population – have many sexual encounters almost every day and changes in knowledge of HIV status can thus lead to observable changes in sexual behaviours nearly immediately. Hence, the four‐month follow‐up period is able to detect the associations of interest, and it is relatively long considering the potential for sexual behaviours to change nearly every day.

However, it is possible that longer‐term effects – for instance, years after a change in knowledge of HIV status – differ substantially from those that evolve over a few months’ time. For example, a FSW who recently learned she was living with HIV might be in shock or feel angry, and thus may not be able or inclined to use condoms to protect her sexual partners from HIV infection. Over time, however, this FSW might come to terms with her HIV status and consequently increase condom use with clients and other sexual partners. Future studies with longer duration of follow‐up should be conducted to gain insights into the effects of HIV status knowledge over several years’ time.

Finally, our results may have limited generalizability. The FSWs who participated in our cohort may have self‐selected on characteristics that affect the relationship between HIV status knowledge and sexual behaviours. Moreover, our associations are limited to participants who changed their knowledge of HIV status over the study period. The latter limitation is unlikely to be severe, however, because the majority of participants (>70%) did change their knowledge of HIV status over the study period. These common changes were likely caused by the increased encouragement and opportunities for HIV testing due to participation in the HIV self‐testing trial.

More broadly, FSW populations across sub‐Saharan Africa are highly diverse 54, 55 and our finding may thus have limited generalizability to other settings, for example, to rural areas or to West Africa, where HIV prevalence in the general population is substantially lower than in East and Southern Africa.

## 5 Conclusions

Frequent HIV testing among FSWs is an HIV prevention strategy recommended by the World Health Organization 66 and implemented by a number of sub‐Saharan African countries. Our study suggests that frequent HIV testing among FSWs may reduce the frequency of sexual risk‐taking behaviours among FSWs who learn they are HIV‐negative, but may increase the frequency of sexual risk‐taking behaviours among some sub‐groups of FSWs who learn they are HIV‐positive. Our results support the expansion of HIV testing programs for FSWs in combination with interventions to ensure rapid linkage to care and HIV treatment initiation for those FSWs who are found to be living with HIV.

## Competing interests

The authors of this study declare no conflicts of interest.

## Authors’ contributions

KFO, MAM, JEH, JAS, CEO and TB conceptualized the paper. KFO conducted the analysis and wrote the first draft. MAM, JEH, JAS, CEO and TB supervised the methodology. KFO, CEO, TN, AN and MC oversaw study administration and the collection of quality data. All authors edited the draft and provided insights into the manuscript.

## Supporting information


**Table S1.** Sensitivity analyses: the effect of knowledge of HIV status on HIV risk‐related sexual behaviours
