# Supplementary material for: Is knowledge of HIV status associated with sexual behaviours? A fixed effects analysis of a female sex worker cohort in urban Uganda
Source: J Int AIDS Soc. 2019 Jul 9;22(7):e25336. doi: 10.1002/jia2.25336 (PMC6615530; doi:10.1002/jia2.25336)
Supplement: Supplementary file 1 — Table S1. Sensitivity analyses: the effect of knowledge of HIV status on HIV risk‐related sexual behaviours [file JIA2-22-e25336-s001.docx]

| **Appendix Table 1. Sensitivity analyses: the effect of knowledge of HIV status on HIV risk-related sexual behaviors** | | | | | |
| --- | --- | --- | --- | --- | --- |
|  |  | **HIV risk-related sexual behaviors** | | | |
|  | **HIV status knowledge^1^** | **Number of clients/**  **average night** | | **Consistent condom use^2^** | |
|  |  | ***IRR*^3^ *(95% CI)*** | ***p*** | ***OR*^3^ *(95% CI)*** | ***p*** |
| ***Sensitivity analysis 1: Poisson and logistic regression models*** | *Unknown* | *ref* |  | *ref* |  |
|  | *HIV-negative* | 0.96 (0.91 to 1.01) | 0.11 | 4.47 (2.27 to 8.78) | <0.001 |
|  | *HIV-positive* | 1.00 (0.94 to 1.06) | 0.95 | 0.76 (0.34 to 1.69) | 0.50 |
|  |  | ***MD*^4^ *(95% CI)*** | ***p*** | ***PP*^4^ *(95% CI)*** | ***p*** |
| ***Sensitivity analysis 2: Unadjusted linear regression models*** | *Unknown* | *ref* |  | *ref* |  |
|  | *HIV-negative* | -0.36 (-0.62 to 0.10) | 0.01 | 0.16 (0.12 to 0.20) | <0.001 |
|  | *HIV-positive* | -0.10 (-0.44 to 0.24) | 0.57 | 0.01 (-0.05 to 0.06) | 0.77 |
|  |  | ***MD*^5^ *(95% CI)*** | ***p*** | ***PP*^5^ *(95% CI)*** | ***p*** |
| ***Sensitivity analysis 3: 10-point HIV status knowledge scale*** | *Very unlikely: 1* | -0.25 (-0.59 to 0.10) | 0.16 | 8.2 (2.1 to 14.2) | 0.008 |
|  | *2* | -0.26 (-0.73 to 0.22) | 0.29 | 13.4 (5.9 to 20.8) | 0.001 |
|  | *3* | -0.64 (-1.02 to 0.26) | 0.001 | 8.7 (3.0 to 14.4) | 0.003 |
|  | *4* | -0.14 (-0.56 to 0.28) | 0.52 | -2.1 (-10.5 to 6.3) | 0.62 |
|  | *5* | *ref* |  | *ref* |  |
|  | *6* | -0.13 (-0.66 to 0.41) | 0.64 | 4.5 (-4.9 to 13.8) | 0.35 |
|  | *7* | -0.60 (-1.10 to -0.09) | 0.02 | -0.6 (-9.8 to 8.5) | 0.89 |
|  | *8* | 0.32 (-0.74 to 0.10) | 0.14 | 0.1 (-8.7 to 8.9) | 0.98 |
|  | *9* | 0.55 (-0.55 to 1.65) | 0.33 | -8.2 (-21.2 to 4.9) | 0.22 |
|  | *Very likely: 10* | -0.24 (-0.71 to 0.23) | 0.31 | -2.0 (-9.8 to 5.7) | 0.60 |
|  |  | ***MD*^5^ *(95% CI)*** | ***p*** | ***PP*^5^ *(95% CI)*** | ***p*** |
| ***Sensitivity analysis 4: Among those who reported testing since start of study*** | *Unknown* | *ref* |  | *ref* |  |
|  | *HIV-negative* | -0.22 (-0.54 to 0.10) | 0.18 | 9.3 (5.1 to 13.6) | <0.001 |
|  | *HIV-positive* | 0.00 (-0.36 to 0.36) | 1.00 | -2.6 (-8.7 to 3.4) | 0.39 |
| **Abbreviations:** female sex worker (FSW), incidence rate ratio (IRR), odds ratio (OR), mean difference (MD), percentage point change (PP), confidence interval (CI), p-value (*p*)  ^1^Participants were asked to report the likelihood they currently had HIV on a 1-10 scale: HIV-negative status (1-3), Unknown HIV status (4-7); HIV-positive status (8-10).  ^2^Categorized as not using condoms with at least one client on an average working night.  ^3^Effect size estimates measured using individual longitudinal data and Poisson (number of clients) or logistic (consistent condom use) regression models with individual effects for individuals, round of data collection, and month; standard errors clustered at the level of the peer educator.  ^4^Effect size estimates measured using individual longitudinal data and regression models with individual effects for individuals; standard errors clustered at the level of the peer educator.  ^5^Effect size estimates measured using individual longitudinal data and regression models with individual effects for individuals, round of data collection, and month; standard errors clustered at the level of the peer educator. | | | | | |
